# Impact and experience of participant engagement activities in supporting dapivirine ring use among participants enrolled in the phase III MTN-020/ASPIRE study

**DOI:** 10.1186/s12889-021-11919-x

**Published:** 2021-11-08

**Authors:** Morgan Garcia, Ellen Luecke, Ashley J. Mayo, Rachel Scheckter, Patrick Ndase, Flavia Matovu Kiweewa, Doreen Kemigisha, Petina Musara, Leila E. Mansoor, Nishanta Singh, Kubashni Woeber, Neetha S. Morar, Nitesha Jeenarain, Zakir Gaffoor, Daniel K. Gondwe, Yvonne Makala, Llewellyn Fleurs, Krishnaveni Reddy, Thesla Palanee-Phillips, Jared M. Baeten, Ariane van der Straten, Lydia Soto-Torres, Kristine Torjesen

**Affiliations:** 1 Global Health Population and Nutrition, FHI, Durham, NC 360 USA; 2 Women’s Global Health Imperative (WGHI) RTI International, Berkeley, CA, USA; ASTRA Consulting, Kensington, CA USA; 3 Population Services International (PSI), 1120 19th St. NW, Washington, DC USA; 4 Makerere University-Johns Hopkins University (MU-JHU) Research Collaboration, Kampala, Uganda; 5 University of Zimbabwe College of Health Sciences-Clinical Trials Research Centre, Harare, Zimbabwe; 6 Centre for the AIDS Programme of Research in South Africa (CAPRISA), University of KwaZulu-Natal, Durban, South Africa; 7 HIV Prevention Research Unit (HPRU), South African Medical Research Council (SAMRC), Durban, South Africa; 8 College of Medicine – Johns Hopkins Bloomberg School of Public Health, Blantyre, Malawi; 9 University of North Carolina Project, Lilongwe, Malawi; 10 Desmond Tutu HIV Foundation, Emavundleni Clinical Research Site, Cape Town, South Africa; 11 Wits Reproductive Health and HIV Institute (Wits RHI), Faculty of Health Sciences, University of the Witwatersrand, Johannesburg, South Africa; 12 Departments of Global Health, Medicine, and Epidemiology, University of Washington, Seattle, WA USA; 13 Center for AIDS Prevention Studies (CAPS), University of California San Francisco, San Francisco, CA USA; 14 National Institute of Allergy and Infectious Diseases, National Institutes of Health, Bethesda, MD USA

**Keywords:** Adherence, Participant engagement, Microbicides, Ring, Dapivirine, PrEP, HIV prevention

## Abstract

**Background:**

Low adherence to investigational products can negatively impact study outcomes, limiting the ability to demonstrate efficacy. To continue advancing potential new HIV prevention technologies, efforts are needed to improve adherence among study participants. In MTN-020/ASPIRE, a phase III randomized, double-blind, placebo-controlled study of the dapivirine vaginal ring carried out across 15 sites in sub-Saharan Africa, a multifaceted approach to adherence support was implemented, including a strong focus on participant engagement activities (PEAs). In this manuscript, we describe PEAs and participant attendance, and analyze the potential impact of PEAs on ring use.

**Methods:**

All sites implemented PEAs and submitted activity and attendance reports to the study management team throughout the study. Participant demographics were collected via case report forms. Residual dapivirine remaining in the last ring returned by each participant was used to estimate drug released from the ring, which was then adjusted for time participants had the ring to calculate probable use categorized into three levels (low/intermittent/high). Product use was connected to PEA attendance using participant identification numbers. We used multivariate Poisson regression with robust standard errors to explore differences in ring use between PEA attendance groups and reviewed qualitative reports for illustrative quotes highlighting participant experiences with PEAs.

**Results:**

2312 of 2629 study participants attended at least one of 389 PEAs conducted across sites. Participant country and partner knowledge of study participation were most strongly associated with PEA attendance (*p* < 0.005) with age, education, and income status also associated with event attendance (*p* < 0.05). When controlling for these variables, participants who attended at least one event were more likely to return a last ring showing at least some use (RR = 1.40) than those who never attended an event. There was a stronger correlation between a last returned ring showing use and participant attendance at multiple events (RR = 1.52).

**Conclusions:**

Our analysis supports the growing body of work illustrating the importance of meaningfully engaging research participants to achieve study success and aligns with other analyses of adherence support efforts during ASPIRE. While causation between PEA attendance and product use cannot be established, residual drug levels in returned rings strongly correlated with participant attendance at PEAs, and the benefits of incorporating PEAs should be considered when designing future studies of investigational products.

## Background

Although the number of new HIV acquisitions each year decreases globally, rates of acquisition remain high among certain populations, including sub-groups of women in sub-Saharan Africa [1]. In order to offer multiple behaviorally congruent, end-user aligned options for HIV prevention that can meet the diverse and evolving needs of these groups and their communities, research to develop new biomedical interventions must continue. Currently, options in the pipeline include daily use and on-demand products as well as longer-acting drug delivery devices [2]. The dapivirine vaginal ring is one such long-acting product, designed specifically for discreet use [3, 4].

In HIV prevention studies, low adherence to investigational products can lead to an inability to evaluate the efficacy of the intervention [5]. This was demonstrated in the FEM-PrEP and MTN-003/VOICE studies of oral tenofovir-based HIV pre-exposure prophylaxis (PrEP) for prevention of HIV among women, where low adherence led to an inability to demonstrate efficacy in these studies, while other studies of the same products with higher adherence showed 49–79% reduction in HIV risk among women [6–8]. Consequently, clinical study teams began to recognize the importance of meaningfully engaging study participants through measures such as enhanced counseling, community sensitization, and group educational and motivational activities, to sustain adherence to study products while in randomized controlled studies [9–12]. Specifically, group activities, including in-person and virtual groups, used to support adherence to medications and positive health behaviors have been shown to increase cohesion, address challenges related to stigma [13], and improve HIV treatment adherence [14], particularly if those activities were flexible and responsive to participant needs [13].

During early implementation in 2012 of MTN-020/A Study to Prevent Infection with a Ring for Extended Use (ASPIRE), it was recognized that strategies to support and encourage high levels of adherence to the study product would be essential to bolster collection of accurate and sufficient data on safety, use, and efficacy of the dapivirine ring. We developed a multifaceted approach to support adherence and engagement by participants, including active community engagement and education, carefully paced accrual, and consideration of participant motivation for enrollment [15]. In addition, participant-centered adherence counseling grounded in strategies for behavior change, continuous monitoring of site-level drug feedback [16], strategies to increase visit retention and accessibility to the ring (such as the conduct of off-site visits, or the provision of extra rings if challenges with visit attendance were anticipated), and enhanced clinic visit efficiency were also implemented. To supplement these strategies, all sites fostered a collective understanding of participant value and contributions to the HIV prevention landscape through the implementation of participant engagement activities (PEAs), defined in ASPIRE as group-level events aimed at promoting study ownership, understanding, and involvement in the study process and product buy-in among participants.

We describe how PEAs were implemented in ASPIRE, explore participant perceptions and feedback related to these activities, and assess the potential effect of PEA attendance on participant ring use during the study.

## Methods

### Study population and design

ASPIRE (NCT01617096) was a phase III randomized, double-blind, placebo-controlled clinical study that enrolled 2629 women ages 18–45 years across 15 clinical research sites (CRS) in Malawi (two sites), South Africa (nine sites), Uganda (one site), and Zimbabwe (three sites) between August 2012 and June 2015. Women were randomized in a 1:1 ratio to use either a monthly silicone elastomer vaginal matrix ring containing 25 mg of dapivirine or a placebo ring containing no drug, and followed monthly for a minimum of 12 and a maximum of 33 months. Study results demonstrated a statistically significant reduction in HIV risk among participants assigned to the dapivirine ring by 27% in intention-to-treat analyses [3], and suggested greater risk reduction among subgroups with evidence of high adherence, exceeding 50% among individuals with consistent ring use [3, 17]. Additional details regarding the study design, recruitment, and results have been published previously [3].

Probes on site-specific PEAs were incorporated into qualitative in-depth interviews (IDIs) and focus group discussions (FGDs) that occurred at six ASPIRE sites, representing each study country and three provinces in South Africa, between February 2013 and June 2015. A total of 214 ASPIRE study participants were recruited with 280 IDIs completed [18].

### Participant engagement activities

Adherence monitoring early in the study indicated the need for increased efforts to improve consistent ring use and adherence [16]. As a result, standard ASPIRE counseling was supplemented with a suite of activities termed “the ASPIRE intervention” that was introduced at study sites 8 months after study initiation, including enhanced counseling, provision of blinded adherence data to site investigators of record, and PEAs [16, 19]. PEAs were site-developed and led, and varied in their design, content, and frequency across the 15 study sites. The study management team encouraged individual site teams to design PEAs to be relevant to their local context and fit within an overall approach to participant engagement that would be flexible and responsive to participants’ changing needs over time. PEAs shared the following common goals: (1) increase participants’ study ownership and personal connection to the research, (2) increase participants’ understanding and awareness of the relationship between product adherence and study outcomes and the impact of individual actions, and (3) increase social incentives for continued study participation through strengthening relationship building among participants, rapport between participants and study staff, and male partner buy-in for participation. In addition, PEAs served as a mechanism to share site-level and study-wide residual drug and pharmacokinetic feedback with participants as well as to promote healthy competition between sites. All study- and product-related educational materials and promotional items shared during events received prior approval from relevant institutional review boards, with community engagement and discussion with key stakeholders also carried out to ensure appropriateness of these items. Investigators and site managers reported quarterly on their sites’ PEAs, providing information on event type, date, location, facilitators, activities/agenda topics, and participant attendance via standardized tracking tools developed by the study management team. Activities and their impact were discussed during regular implementation calls between sites and the study management team. Sites also discussed outcomes of and their experiences with PEAs via presentations during meetings with the full protocol team.

### Measures

As defined above, PEAs were group events carried out with the intent of building study ownership, understanding, participant agency, and involvement in the study process and improving product buy-in among participants. In post-hoc analysis, PEAs were categorized into one of five categories: social events; adherence workshops; male involvement events; couple events; and annual or semi-annual events as described in the results section.

*Attendance at PEAs:* The number of PEAs attended by each participant was summarized from PEA reports submitted by the sites and categorized as 0 or 1+ event(s). Male partner attendance was summarized based on participant self-report on the study exit visit Case Report Forms (CRF).

*Sociodemographic characteristics:* Demographic and behavioral characteristics data was collected via CRF for all study participants, including: participant study site, age (18–21, 22–26, and 27–45 years), marital status, educational attainment level (“secondary school complete or more” or “secondary school not complete or less”), whether the participant earned an income outside of the home, travel distance to the clinic (< 1 h, ≥ 1 h), and male partner knowledge of their study participation.

*Ring Use:* Phase I studies established that on average > 4 mg of dapivirine is released from the ring with consistent use for 28 days [20]. As described elsewhere [3], residual drug levels were used to estimate the amount of drug released per month and adjusted for time participants had the ring were divided into three categories of ≤0.9 mg (no or very low use), 0.9–4 mg (intermittent use), and > 4 mg (high use). Ring refusal by participants was treated as time not used, and rings that were not returned by participants were categorized as unused. Plasma samples were also collected quarterly and tested for dapivirine as a measure of product use. However, this measure is representative of use in the previous 8 h and is not indicative of continuous use over the period between study visits [21], and therefore was not utilized as the ring use outcome in this analysis.

### Analysis

We used descriptive statistics to summarize sociodemographic characteristics and behavior of participants by PEA attendance category (0 or 1+ participant engagement activity event [s]). To explore whether sociodemographic characteristics differed between those who did not attend any events and those who attended at least one event, we used a Poisson regression model with robust standard errors including the following data points: country, age, marital status, educational attainment level, earning an income outside of the home, travel distance to clinic, partner awareness of study participation, and study arm. Ring use was reported based on analysis of drug released from the last ring returned by a participant, stratified by PEA attendance category. To examine whether ring use differed between the PEA attendance groups, we used a robust Poisson model, controlling for country and the number of months the participant had been in the study to-date. The additional sociodemographic characteristics listed above were also tested within the same model. In this analysis, South Africa was selected as the reference group because it had the largest number of participants.

### Qualitative analysis

Qualitative interviews conducted throughout ASPIRE included probes about participant experiences with PEAs as part of the IDIs: “*Tell me about your experience being part of ASPIRE*” and “*What could we (do/have done) to improve your experience in the study?*” and the FGDs: *“What were participants’ attitudes towards workshops, meetings, and other social activities at the clinic?”* A themed analysis of qualitative data related to PEA participation was conducted by reviewing code reports, stratified by site, for data assigned with the parent code ACTIVITIES. Quotes highlighting participant experiences with engagement events are presented in the Results section. Irrelevant text within quotations has been removed and replaced with “…” , and clarifying text is included within parentheses.

## Results

### Participant engagement activities

A total of 389 PEAs were carried out by the 15 ASPIRE sites throughout the study, reaching a total of 2312 of 2629 participants. Engagement activities fell into five main categories: adherence workshops, social events, annual and semi-annual events, male involvement events, and couple events. Adherence workshops for participants represented the majority of events (*n* = 251; 64.5%) conducted by sites, with both social and annual or semi-annual events each representing 10.5% (*n* = 41) of events, and a small portion of activities dedicated to male partner engagement (*n* = 34; 8.7%) and couple events (*n* = 22; 5.7%). Table 1 provides descriptions and frequency of each activity type by country and city or province.

**Table 1:** Description and frequency of PEAs and participant attendance by country and city/province

Country, City/Province	Social events	Adherence workshops/ Sessions	Male involvement	Couples events	Annual/semi-annual events	Total events at site	Participant attendance at one or more events
	N (%)	N (%)	N (%)	N (%)	N (%)	N (%)	N/N (%)
**Total**	**41 (11)**	**251 (65)**	**34 (9)**	**22 (6)**	**41 (11)**	**389**	**2312/2629 (88)**
**South Africa (SA)**	**41 (19)**	**105 (48)**	**15 (7)**	**22 (10)**	**35 (16)**	**218**	**1152/1426 (81)**
SA – Cape Town (1 site)	7 (27)	19 (73)	–	–	–	26	130/166 (78)
SA – Johannesburg (1 site)	3 (6)	34 (72)	1 (2)	6 (13)	3 (6)	47	161/213 (76)
SA – Durban (CRS 1) (1 site)	13 (35)	10 (27)	11 (30)	–	3 (8)	37	222/244 (91)
SA – Durban (CRS 2) (6 sites)	18 (17)	42 (39)	3 (3)	16 (15)	29 (27)	108	639/803 (80)
**Uganda –** **Kampala** (1 site)	–	**36 (100)a**	**–**	**–**	**–**	**36**	**246/253 (97)**
**Malawi**	**–**	**38 (75)**	**13 (25)**	**–**	**–**	**51**	**252/272 (93)**
Malawi **–** Lilongwe (1 site)	–	22 (81)	5 (19)	–	–	27	134/142 (94)
Malawi **–** Blantyre (1 site)	–	16 (67)	8 (33)	–	–	24	118/130 (91)
**Zimbabwe – Harare** (3 sites)	**–**	**72 (86)**	**6 (7)**	**–**	**6 (7)**	**84**	**662/678 (98)**

Bolded text highlights totals by country and across the study

^a^The Kampala site included workshops focused on male involvement within the category of “adherence workshops”

#### Adherence workshops

Activities conducted at adherence workshops ranged from general discussions on the community impact of HIV, prevention of sexually transmitted infections, and the use of long-acting reversible contraceptive (LARC) methods, to study product-related conversations, including sharing personal ring use experiences as well as challenges and concerns. These events provided an opportunity to give participants tokens of appreciation – such as toiletry bags, household goods, or makeup – for their continued commitment to study participation and visits, while also engaging them in educational topics. Participants across research sites reported that hearing messages about the ring and HIV prevention from study staff in a group setting positively changed their behavior and helped them feel more equipped to adhere to the study requirements. As one participant in Kampala stated:

Before we came to the meeting, some of us used to remove the rings, wash them, and re-insert them, wondering how we were to return them dirty to the study clinic. What helped us was that each one had to tell the truth and for example say: “Health worker, for me I removed it, washed it, and re-inserted it.” Then they (study staff) told us; “No, you should leave it inserted. It has no problem. You should remove it from here (at study clinic). That made us change but we had that habit (of removing ring, washing it, and then re-inserting it)*.*

(30-year-old, FGD, Kampala)

Similarly, sharing ring experiences also helped participants overcome fears and feel motivated to continue with the study participation and ring use.

They were even talking about the problems that they are having with the rings, I liked that session we had, because they did things I could relate to, a person would advise you like I did this and that and you too will find that, do you see? You get advice from one another*.*

(22-year-old, FGD, Cape Town)

Events themselves are important as we motivate each other about using the ring. People say different things and share their opinions, they also share how the ring makes each of them feel, others tell us why they remove the ring. Some participants share their challenges with the ring, whereas others share different experiences with the ring. We learn a lot and teach each other a lot from these events*.*

(25-year-old, IDI, Durban)

During adherence workshops, participants were able to process their motivations for joining the study more deeply, including their personal experiences with HIV and AIDS. Through these discussions, study staff supported participants to link their altruistic motivations to an understanding of the importance of adherence to the study product. As one participant in Johannesburg noted:

Okay, another participant really touched me. She grew up in a place … I can’t remember where but it was outside South Africa. She said her mother was abused by her stepfather. One day the nursing sisters came into their house and gave her mother condoms. When her mother showed her stepdad the condoms, he beat her. He asked her “What is this for?”. The man was very abusive. So she (the participant who related the story) said that she is doing this (participating in the study) to empower other women in (the) future because women … would be able to protect themselves against HIV because some partners don’t want to use condoms yeah*.*

(23-year-old, IDI, Johannesburg)

These workshops also presented an opportunity for site staff to emphasize the impact of adherence to the ring on study outcomes, share site-level adherence feedback, normalize adherence challenges, dispel rumors related to the study and study product, and to discuss the future of the dapivirine ring in the event of positive study results.

Participants reported feeling a sense of ownership of and responsibility to the study site and their fellow participants after learning about site-level adherence feedback. One woman in Uganda said:

What I like about it, maybe, I want to be persistent in using the ring, so that I can get what I am fighting for: maybe it will prevent [HIV acquisition]. The other day, we were in a meeting and we tried to encourage ourselves that those who use it and those who do not should persist and continue with the ring*.*

(26-year-old, IDI, Kampala)

Other participants felt supported and adopted strategies to manage community rumors after participating in events:

You know it’s possible for somebody to have challenges along the way as I did with my neighbors; they were talking bad about this study. But when we met during these adherence meetings, each one of us talks about the challenges we encounter according to how you faced them and how you resolve them using the expertise we gather from these adherence meetings*.*

(24-year-old, IDI, Lilongwe)

Finally, in Zimbabwe, participants expressed hope for what the ring could mean for the future of HIV prevention:

I mentioned before that a lot of us women did not have the ‘power’ over condoms so a lot of people like that if the ring is found effective it will be something that will help a lot of women*.*

(28-year-old, IDI, Harare)

#### Social, annual, and semi-annual events

Through social, annual or semi-annual events (*n* = 82; 21%), sites engaged participants in a wide range of activities, such as tea parties, cupcake decorating contests, skills training, movie viewings, holiday celebrations, and even a “Miss ASPIRE” pageant. These events, while not directly addressing adherence, provided an opportunity for participants and site staff to build rapport and trust. They also gave participants a chance to relax, have fun, and develop relationships with each other—with reserved participants gaining comfort with social interactions and confidence to be more open during other engagement events, including adherence workshops.

Participants reported enjoying the social events as a time to interact and build relationships with one another and staff, learn new skills, and showcase their talents:

I like the events because we get an opportunity to meet together without having to hear about blood draws, injections and the product. But important things are still addressed in such events though it is not the focus of the meeting. They want us to feel relaxed. Participants express their feelings in these events, we share our views and hear each other’s opinions*.*

(20-year-old, FGD, Durban)

#### Male involvement events and couples events

Male involvement events and couples events (*n* = 56; 14.4%) were conducted to increase male partner understanding of the dapivirine ring and the ASPIRE study, to address negative rumors, and generally build male partner support for study participation and use of the ring. Based on ASPIRE participant self-report at the study exit visit, 263 male partners (12%) attended one or more events. These events specifically addressed male partner concerns about the ring, including its safety during sexual intercourse, and provided answers to questions about HIV prevention and transmission, as well as other health topics like family planning. During some of these events, male partners were offered on-the-spot HIV counseling and testing and made appointments for voluntary medical male circumcision. To protect confidentiality during these voluntary events, pseudonyms were used, and all ASPIRE participants received individual counseling and waiting room education on male partner engagement to ensure that they were aware of topics to be discussed to minimize the potential for social harm and enable participants to make informed choices.

Participants expressed appreciation for male partner events, saying that hearing the information from study staff—and seeing that many women were participating in ASPIRE—helped calm male partner fears about study participation and ring use:

When we came with men those who were free to come with their husbands... I found it helpful because ‘even’ for the men we came with, some will not really understand when you alone explain to him, him looking at the papers that you go home with but when you come here with him he will get enough knowledge. He will see that a lot of women are in ASPIRE.... So I find it helpful in that even if you tell him tomorrow (in future) that I am going to … (the study site) to ASPIRE... It will not scare him because he would have come to see what will be taking place, hearing from those who are trained*.*

(28-year-old, IDI, Harare)

### Participant characteristics by PEA attendance

Based on site attendance records, 88% of participants attended at least one participant engagement event, with 74% attending two or more events. Event attendance varied by site and by country, with 81% (range 76–91%) of participants attending an event across all South African sites versus 93% (range 91–94%) in Malawi, and 98% in Uganda and Zimbabwe (*p* < 0.0001).

Demographic characteristics of participants by PEA attendance are presented in Table 2. In a multivariate robust Poisson regression analysis of PEA event attendance, country, education, earning an income outside of the home, and partner awareness of study participation were significantly associated with attending at least one event. Country was the strongest predictor of event attendance, with participants in Malawi, Zimbabwe, and Uganda being more likely to attend at least one event compared to participants in South Africa (relative risk [RR] 95% Confidence Intervals [CIs]: 1.13 [1.07–1.19], 1.20 [1.16–1.25], and 1.27 [1.20–1.35], respectively). Participants who had told their partners about study participation were 9 % more likely to have attended an event compared to those who did not tell their partners (RR, 95% CI: 1.09 [1.04–1.15]). Participants ages 22–26, those who completed secondary school or more, and those who earned an income outside of the home were less likely to have attended an event (RR, 95% CI: 0.95 [0.91–0.99], 0.97 [0.94–0.99], 0.97 [0.94–0.99], respectively).

**Table 2:** Demographic characteristics of participants by site-reported PEA attendance

	PEA event attendance		Poisson regression of PEA event attendance	
	0 events	1+ event		
	**N (row %)**	**N (row %)**	**RRa (95% CI)**	**P**
N	317	2312	2628	
Country				
Malawi	20 (7)	252 (93)	1.13 (1.07–1.19)	< 0.001
South Africa	274 (19)	1152 (81)	Ref	
Uganda	7 (3)	246 (97)	1.27 (1.20–1.35)	< 0.001
Zimbabwe	16 (2)	662 (98)	1.20 (1.16–1.25)	< 0.001
Age				
18–21	71 (14)	451 (86)	Ref	
22–26	120 (14)	723 (86)	0.95 (0.21–0.99)	0.029
27–45	126 (10)	1137 (90)	0.97 (0.93–1.01)	0.161
Married	52 (5)	1022 (95)	1.01 (0.98–1.05)	0.469
Secondary school complete or more	179 (15)	1020 (85)	0.97 (0.94–0.99)	0.029
Earns income outside of home	144 (12)	1042 (88)	0.97 (0.94–0.99)	0.021
Travel distance: 1 h or more	62 (9)	604 (91)	0.97 (0.94–1.01)	0.126
Partner knows you are participating in the study	232 (11)	1869 (89)	1.09 (1.04–1.15)	< 0.001
Study Arm: Active	167 (13)	1146 (87)	0.98 (0.96–1.01)	0.268

^a^Multivariate model includes all demographic characteristics listed in the table

#### Ring use and PEA attendance

**Fig. 1:**
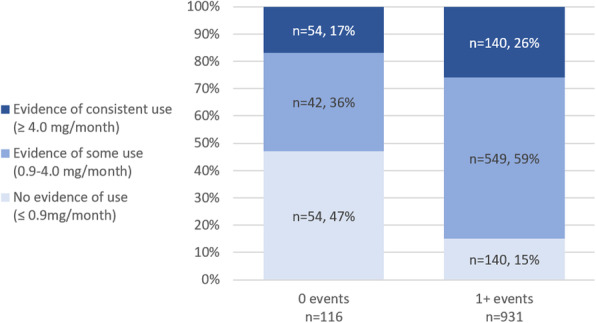
Proportion of last returned rings in each ring use category by site-reported PEA attendance

Ring use was defined by the amount of drug released from the last ring a participant returned as a proxy for persistent use. Among participants in the active arm of the study who had at least one returned ring evaluated for drug release (*n* = 1036), 83% returned a last ring which showed evidence of at least some use (> 0.9 mg drug release). Eighty-five percent of women who attended one or more events returned last rings showing evidence of at least some use, compared to 53% of women who did not attend any events (Fig. 1).

#### Ring use category of participants by site-reported PEA attendance

In univariate and multivariate Poisson regression analyses with robust standard errors, some or high ring use among participants in the active study arm was associated with event attendance independent of sociodemographic characteristics or country. Table 3 presents the results of multivariate robust Poisson regression analyses of use controlling for participant sociodemographic and behavioral characteristics and the duration of time a participant had been in the study when her ring was returned. Model 1 shows a significant association between attendance at least one event and having the last returned ring indicate at least some use, compared with attendance at no events (RR = 1.48, 95% CI [1.25–1.77]), *p* < 0.001). Country and time in the study when the ring was returned were the only other significant correlates with ring use. Women participating in Malawi, Uganda, and Zimbabwe were 12–14% more likely to return a ring indicating some usewhen compared to women in South Africa (RR [95% CIs]: 1.14 [1.05–1.24], 1.13 [1.00–1.28], 1.12 [1,01–1.25], respectively). The relative risk of the last returned ring indicating some use increased with each month that the participant had been enrolled in the study when the ring was returned (RR = 1.01, 95% CI [1.00–1.01], *p* = 0.032).

**Table 3:** Multivariate robust Poisson regression of use for last returned ring

	Poisson regression of ring use (> 0.9 mg of drug released)	
	RR^**a**^ (95% CI)	P
N	1046	
Model 1:		
Attended 1+ events	1.48 (1.25–1.77)	< 0.001
Model 2:		
Attended 1 event	1.40 (1.15–1.70)	0.001
Attended 2+ events	1.52 (1.27–1.81)	< 0.001

^a^Multivariate model includes follow-up months and sociodemographic characteristics listed in Table 2 (country, age, marital status, educational attainment, earns an income, travel distance, and partner knowledge of study participation)

When event attendance was broken down further into attendance at 0, 1, or 2+ events in Model 2, women who attended an event once were 40% more likely to have the last returned ring indicate at least some use (RR = 1.40, 95% CI [1.15–1.70], *p* = 0.001) compared to those who did not attend any events, while those who attended two or more events were 52% more likely to have returned a ring that had been used (RR = 1.52, 95% CI [1.27–1.81], *p* < 0.001).

## Discussion

We implemented and evaluated the use of participant engagement activities during a phase III clinical study of the dapivirine vaginal ring for HIV prevention. At the time of study implementation, there were no published reports of this type of group events utilized to support participant adherence in HIV prevention trials. PEAs were therefore broadly implemented, measured, and reported on for the first time in the ASPIRE trial to encourage a sense of study ownership among participants, break down barriers and power imbalance between staff and participants, and create a supportive environment for providing education and learning from participants, and sharing of experiences in a group setting. These were all seen to be avenues for improving participant understanding of their study participation and adherence to study product.

Study sites demonstrated that PEA implementation was feasible, with all sites successfully organizing between 14 and 47 events and nearly 90% of participants attending at least one event based on site report. Although participants were reimbursed for transport to attend PEAs across sites, attendance varied by country. While nearly all participants in Uganda and Zimbabwe attended at least one event, participants in South Africa were less likely to have attended any events. There is limited data to explain the difference in attendance, but this could be due to variability in the participant population by site that were not measured during the study, differences in the relationship dynamics between staff and participants, or varied site resources dedicated to PEAs. However, even sites with lower event attendance still reached 75% of participants with PEAs over the course of the study. It is possible that women whose partners were unaware of their study participation were less likely to attend PEAs; events were often held outside of regular study visits and these participants may have had challenges related to leaving home or employment to attend. Participants with higher education levels and who earned an income of their own were also less likely to attend PEAs, potentially due to time constraints from work, school, or other obligations. These women may have prioritized their time to attend ASPIRE clinic visits rather than optional engagement events.

In qualitative interviews, participants spoke about how PEAs impacted their product adherence and fostered a sense of ownership in the study. Few participants had neutral or negative feedback regarding the events; those who did mainly expressed disappointment that they were not able to attend events due to work or other personal commitments. While remote engagement platforms, such as WhatsApp or social media groups, were not used for group engagement during this study, these could present a means for engaging with participants who are unable to attend in-person events due to employment or other commitments in future studies.

While the PEAs took place within a broader study context where adherence support was a priority, the group nature of PEAs helped participants feel reassured about the study product and motivated to use it. PEAs created a collaborative environment where participants and staff became part of a team working toward a common goal, each with responsibilities to the other; site staff communicated openly about study progress and site-level adherence feedback, and participants spoke freely about adherence successes and challenges, community rumors, and male partner perceptions of study participation.

Our analysis of PEA attendance and the estimated drug released based on residual drug data in the last ring returned by participants illustrates that participants who attended at least one event were more likely to have used the study product at least some of the time during the last month of ring use. Furthermore, we observed a dose response wherein the participants who attended two or more events were even more likely to return a last ring indicating at least some use. Group engagement events allow for different types of interactions between participants and research staff than one-on-one interventions, and our research suggests they may be an important tool that can be used to boost product use in clinical studies. Other analyses of the ASPIRE intervention have also concluded that the PEA approach contributed to reducing participant concerns related to ring use and increased feelings of altruism regarding their ASPIRE study participation, and was associated with increased ring use during the study [19, 22].

Findings from this analysis align with findings from similar interventions carried out during and after the ASPIRE study. Studies of antiretroviral therapy (ART) adherence clubs implemented in South Africa, for example, reveal that these clubs have a positive impact on women’s correct and consistent use of ART, as well as their self-reported mental health [[23, 24]].

Our analysis has several limitations, the first of which is that residual drug levels in returned rings were not captured at the start of the study, making it difficult to determine objective levels of ring use pre- and post-PEA implementation. While we found a strong correlation between the drug levels in last rings returned by participants and attendance at PEA events, causation cannot be established. We included only the last returned ring to avoid issues of temporality of event attendance and to assess persistence of ring use, which may be a proxy for longer-term use behavior after attending events. Although PEA attendance was assessed by site report, as well as self-report by participants on end-of-study CRFs, site-reported PEA attendance data was used for this analysis because it was collected in real time, rather than via retrospective self-report, and captured a larger proportion of study participants. Our use of site-reported data allows for inclusion of the 17% of participants who did not complete a study exit visit, avoids the over-representation of ring users in our analysis, and avoids the social desirability and recall biases that might be present in self-reported data from participants. However, there is the potential for measurement error in the chosen data source due to human error and the use of site-maintained documents rather than a CRF. In addition, PEAs were not standardized across sites; while this allowed each site to be flexible to the needs of their specific participant population, this complicates our ability to pinpoint which approaches were the most effective or generalize across the study. Other analyses have found additional factors that were correlated with adherence, such as use of LARCs and frequency of menses, but these were not included in our analysis as it is unlikely that they would impact event attendance (Husnik, Correlates of Adherence, under review).

## Conclusions

Conducting PEAs spanning a range of approaches, from purely social and recreational events, shared storytelling of the community impact of HIV, to study- and product adherence-focused discussions, was an acceptable and effective approach to supporting dapivirine ring use among participants in the ASPIRE study. The PEA approach should be considered as a method for increasing participant ownership in other clinical studies where high adherence is necessary for achieving study goals. Engagement events may have a cumulative effect that can greatly enhance adherence for participants who attend multiple events.

Based on the experience of the ASPIRE study team, group engagement activities are a low-cost approach that can complement more focused adherence support strategies. These offer a flexible and context -specific activities that provide participants with opportunities to share experiences in an environment that encourages mutual support.

This analysis contributes to the growing body of work illustrating the importance of participant buy-in and engagement, specifically through group education and support activities, during study implementation. More research is needed to investigate the specific mechanism by which PEAs may contribute to improved adherence, in order to allow for streamlining and efficiency in implementation while preserving the flexibility and adaptability of the approach. Ultimately, we strongly recommend that similar participant engagement activities be built into other HIV prevention studies, particularly studies of investigational products or drugs, and any study seeking improved adherence to study products.

## Data Availability

All data generated or analyzed as part of the MTN-020/ASPIRE trial are included in this article, Use of a Vaginal Ring Containing Dapivirine for HIV-1 Prevention in Women, and its supplemental files. Additional data related to implementation of participant engagement events at ASPIRE sites is available from the corresponding author upon reasonable request.

## Abbreviations

ART: Anti-retroviral therapy
ASPIRE: A Study To Prevent Infection with a Ring for Extended Use
CI: Confidence Interval
CRF: Case Report Form
HIV: Human Immunodeficiency Virus
LARC: Long-Acting Reversible Contraceptives
MTN: Microbicide Trials Network
PEA: Participant Engagement Activity
RCT: Randomized Controlled Trial
RR: Risk Ratio
SA: South Africa
VOICE: Vaginal and Oral Interventions to Control the Epidemic
